# Enhanced anti-herbivore defense of tomato plants against *Spodoptera litura* by their rhizosphere bacteria

**DOI:** 10.1186/s12870-022-03644-3

**Published:** 2022-05-24

**Authors:** Sumei Ling, Yi Zhao, Shaozhi Sun, Dong Zheng, Xiaomin Sun, Rensen Zeng, Dongmei Chen, Yuanyuan Song

**Affiliations:** 1 Key Laboratory of Ministry of Education for Genetics, Breeding and Multiple Utilization of Crops, College of Life Sciences, Fujian Agriculture and Forestry University, Fuzhou, China; 2 Institute of Crop Resistance and Chemical Ecology, College of Agriculture, Fujian Agriculture and Forestry University, Fuzhou, 350002 China

**Keywords:** *Stenotrophomonas rhizophila*, Rhizosphere bacterium, Tomato, *Spodoptera litura*, Anti-herbivore defense

## Abstract

**Background:**

The use of beneficial microorganisms as an alternative for pest control has gained increasing attention. The objective of this study was to screen beneficial rhizosphere bacteria with the ability to enhance tomato anti-herbivore resistance.

**Results:**

Rhizosphere bacteria in tomato field from Fuqing, one of the four locations where rhizosphere bacteria were collected in Fujian, China, enhanced tomato resistance against the tobacco cutworm *Spodoptera litura*, an important polyphagous pest. Inoculation with the isolate T6–4 obtained from the rhizosphere of tomato field in Fuqing reduced leaf damage and weight gain of *S. litura* larvae fed on the leaves of inoculated tomato plants by 27% in relative to control. Analysis of 16S rRNA gene sequence identities indicated that the isolate T6–4 was closely related to *Stenotrophomonas rhizophila* supported with 99.37% sequence similarity*.* In the presence of *S. litura* infestation, inoculation with the bacterium led to increases by a 66.9% increase in protease inhibitor activity, 53% in peroxidase activity and 80% in polyphenol oxidase activity in the leaves of inoculated plants as compared to the un-inoculated control. Moreover, the expression levels of defense-related genes encoding allene oxide cyclase (*AOC*)*,* allene oxide synthase (*AOS*)*,* lipoxygenase D (*LOXD*) and proteinase inhibitor (*PI-II*) in tomato leaves were induced 2.2-, 1.7-, 1.4- and 2.7-fold, respectively by T6–4 inoculation.

**Conclusion:**

These results showed that the tomato rhizosphere soils harbor beneficial bacteria that can systemically induce jasmonate-dependent anti-herbivore resistance in tomato plants*.*

**Supplementary Information:**

The online version contains supplementary material available at 10.1186/s12870-022-03644-3.

## Key message

Rhizosphere bacterium *Stenotrophomonas rhizophila* enhances tomato resistance against *Spodoptera litura* by systemic induction of jasmonate-mediated defense.

## Background

Tomato (*Solanum lycopersicum* Castlemart) is one of the most consumed fruits and vegetables worldwide due to its richness in dietary fiber, carbohydrates, vitamins A and C, minerals such as boron, phosphorus and manganese [1]. Due to its health benefits, the tomato is also processed into various convenient products including juice, ketchup, sauce and tomato soups. However, tomato plants are subjected to a variety of biotic and abiotic stresses in the whole development stages from seedling to fruit-bearing. It suffers from severe damage by many insect pests including aphids and white fly (*Bemisia tabaci* Genn) [2]. The tobacco cutworm *Spodoptera litura* (Lepidoptera: Noctuidae), a highly polyphagous pest of many important crops, also causes significant tomato yield losses [3]. Therefore, novel approaches of controlling *S. litura* are urgently needed in the agricultural production.

Traditional agricultural practices for the control of *S. litura* involve the use of insecticides [4]. Although the application of insecticides is considered to be an effective control strategy the insect pests can rapidly develop high level of resistance upon the extensive and overdose use of insecticides [5, 6]. Insecticide resistance in turn substantially increases the worldwide application of insecticides. Consequently, it severely threatens efficient pest management [7, 8]. Hence, the use of the biological control strategy for tomato protection against insect pests is considered as an environmentally friendly option.

Beneficial microorganisms as an alternative to agro-chemical application for crop protection against microbial pathogens and insect attackers have been extensively studied in recent years and has become an increasingly important method. The positive impacts of beneficial microorganisms with the advantages of the growth promotion and enhanced tolerance to the biotic factors have been studied in many plants such as tomato [9], pepper [10], maize [11] and cotton [12]. It has been shown that many plant growth-promoting rhizobacteria (PGPR) colonized at the soil-root interface have great potential for improving crop productivity by phosphate solubilization, nitrogen fixation, or disease suppression [13, 14]. Most importantly, some PGPR could induce systemic resistance (ISR) in host plants [15]. Plant defense against insect attack is associated with various phytohormones. Jasmonic acid (JA) is the key phytohormone that mediates plant defense against herbivores [16–18]. The expression of JA signaling pathway related genes *GhAOS, GhLOX1* and *GhOPR3* in cotton can be induced after inoculation with beneficial rhizosphere microorganisms [12]. The JA signaling pathway has been demonstrated to play a crucial role in rhizobacteria-triggered ISR of *Arabidopsis thaliana* against the generalist caterpillar *Mamestra brassicae* [19]. In addition, studies have shown that PGPR-mediated ISR is often associated with enhanced expression of plant responsive genes encoding proteinase inhibitors (PIs) and with increased activities of defense-related enzymes [20–22]. It has been well documented that PIs are crucial for plant defense against insect herbivores [23, 24]. Upon insect attack, defense responses can be induced more rapidly in PGPR-inoculated plants. For example, inoculation of cotton plants with *Bacillus* spp. can rapidly induce accumulation of JA and increase transcript level of JA responsive genes [12]. Inoculation of tomato plants with *Bacillus subtilis* induces systemic resistance against gray mold [25] and whitefly *Bemisia tabaci* [26].

The aim of the study was to screen the beneficial rhizosphere bacteria with the potential to improve tomato resistance against the polyphagous pest *S. litura.* The T6–4 isolate defined as *Stenotrophomonas* sp. was successfully obtained and its capacity to induce tomato ISR was evaluated by examining the defense-related enzymes, genes and proteinase inhibitor.

## Materials and methods

### Tomato plants and *Spodoptera litura*

Tomato seeds (*Solanum lycopersicum* Castlemart; TGRC accession: LA2400) were provided by Prof. Chuanyou Li of the Institute of Genetics and Developmental Biology, Chinese Academy of Sciences. They were surface sterilized for 5 min in 10% H_2_O_2_, rinsed with sterilized distilled water for three times, and then germinated on autoclaved soil beads according to the method described by Song et al. [27]. Ten-days-old tomato seedlings were transplanted to pots for further experiments.

The caterpillar *Spodoptera litura* population was provided by the Institute of Entomology, Sun Yat-sen University (Guangzhou, China). Its larvae were fed on the semi-synthetic diet as described by Gupta et al. [28] and maintained in an insectary (23–26 °C, 65–70% relative humidity) in the laboratory.

### Chemicals

The Bacterial Genomic DNA Extraction UNIQ-10 kit was obtained from Sangon Biotech (Shanghai, Co., Ltd. China). SYBR Green Real-time PCR Master Mix was purchased from Toyobo Life Science (TOYOBO Co. Ltd., OSAKA, Japan). The other chemicals used in the study were purchased from Sigma-Aldrich (St. Louis, MO, USA).

### Rhizosphere soil sampling for screening bacteria that induced tomato defense

Rhizosphere soil samples were collected from tomato fields in Fuqing, Fuzhou, Putian and Minhou in Fujian province (Southeast China). The sampling method was performed according to the methods previously described [11, 29]. Briefly, soil samples were randomly collected from rhizosphere soil of tomato plants in four locations. The collected rhizosphere soils were passed through a 4 mm sieve to eliminate plant materials, then the prepared soil samples were stored at 4 °C until further use.

To examine the effect of rhizosphere bacteria on tomato resistance against the chewing caterpillar *S. litura* the soil slurry was prepared as described by Kostenko et al. [30] and Yuan et al. [31] with slight modification. Fifty grams of each soil sample were mixed with 500 mL sterile distilled water and incubated at room temperature overnight, then the soil slurry was obtained by filtering through a Whatman No. 42 filter-paper. Ten-days-old tomato seedlings were transplanted into pots filled with 2 kg autoclaved soil/sand mixture (2:1) and inoculated with 50 mL soil slurry obtained from rhizosphere soils from Fuqing, Fuzhou, Putian and Minhou, respectively, or 50 mL autoclaved water as control. Each treatment contained 10 tomato plants that were randomly placed in the greenhouse with 21/16 °C day/night. The plants were irrigated with sterile water twice a week for 30 d. Then, the larvae with similar body weights were transferred on tomato leaves with sterile tweezers (3 larvae per plant) and the tomato plants were covered with a breathable mesh bag to prevent pests from escaping. Thirty *S. litura* larvae were used for each treatment. The weight gains of *S. litura* larvae were recorded 48 h after larval inoculation.

### Isolation and screening of bio-control bacteria

Tomato rhizosphere soil samples from Fuqing were carefully collected by uprooting the root system and shaking the loose soil around the roots. The obtained rhizosphere soil was kept at room temperature for air-dry. Five grams of the obtained rhizosphere soil were added into a 150 mL Erlenmeyer flask containing 50 mL of sterile distilled water. After shaking the flask at 120 rpm for 30 min, the suspension was used to isolate associated rhizosphere bacteria by serial dilutions method [32]. Then, 100 μL aliquots (10^− 6^ to 10^− 4^) were spread on plates with Tryptic soy agar (TSA), Lysogeny broth (LB) or Nutrient agar (NA) (Table S1). The agar plates were incubated at 28 °C till the bacterial colonies appeared on the plates. Morphologically different colonies were isolated and purified using the serial dilution plating technique [33]. All isolates were stored at − 80 °C for further analysis.

To screen the bio-control bacteria with the ability to enhance the tomato plant resistance against *S. litura,* ten-days-old tomato seedlings were transplanted into the pots as described above. The bacterial suspension of isolate (OD600 ≈ 0.8) was collected and added to the soil around tomato roots. An equal volume of distilled water was injected into the other group to serve as the control. For each treatment, 20 tomato plants were inoculated and randomly placed in the greenhouse with 21/16 °C day/night. Thirty days after bacterial inoculation the plants were inoculated with 3rd-instar larvae on the leaves (3 larvae per plant), then covered with a breathable mesh bag to prevent pests from escaping. Sixty *S. litura* larvae were used for each treatment. The weight gain of *S. litura* larvae in 72 h was used as an indicator of plant anti-herbivore resistant level.

### Identification of bacterial T6–4 isolate

Colony morphology, gram staining and 16S rRNA gene sequence were carried out to identify the bacterial T6–4 isolate that induced tomato anti-herbivore resistance. T6–4 isolate was cultured in TSA culture medium and incubated at 28 °C for 2 days, and then the colony morphology was then observed with naked eyes. Subsequently, gram staining was carried out to further identify the T6–4 isolate. In addition, 16S rRNA gene sequence was conducted to confirm the identification of T6–4 isolate [34, 35]. Bacterial genomic DNA was extracted and purified using a Sangon Bacterial Genomic DNA Extraction UNIQ-10 kit (http://www.sangon.com/, China). Then, polymerase chain reaction (PCR) was performed to amplify the partial 16S rRNA gene of T6–4 isolate using primers 27F (5′-AGAGTTTGATCCTGGCTCAG-3′) and 1492R (5′-GGTTACCTTGTTAC GACTT-3′). The DNA generated by PCR was sequenced by Sangon Biotech (Shanghai) Co., Ltd. A homology search of the related 16S rRNA gene sequences was performed using BLAST search against the nucleotide database (https://blast.ncbi.nlm.nih.gov/Blast.cgi). Alignment of the related 16S rRNA gene sequences was performed and a phylogenetic tree was constructed using MEGA 5.0 according to the neighbor-joining (NJ) method with 1000 bootstrap replications.

### Determination of the content of protease inhibitor (PI) and activities of defense-related enzymes in tomato leaves

The ELISA kit was used to determine the activity of PI in tomato leaves according to the instruction of the kit. Activities of peroxidase (POD) and polyphenol oxidase (PPO) were detected according to the reported methods with slight modification [27, 36, 37]. Three days after insect inoculation, leaf samples (100 mg, fresh weight) from tomato plants un-inoculated or inoculated with T6–4 isolate were collected and ground to a fine powder in liquid nitrogen. Then, samples were homogenized in phosphate buffer (0.05 M) containing 1% (w/v) polyvinylpyrrolidone (PVP), and the optimum pH of the buffer was adjusted to 7.2 for POD and 7.8 for PPO. The supernatant was obtained after centrifugation at 10000 rpm at 4 °C for 15 min and used for analysis of defense-related enzyme activity. The reaction was initiated by adding supernatant of POD and PPO extract and the change in absorbance at 470/525 nm was recorded. The enzyme activities were calculated as the units of enzyme activity per mg of protein. Three replicates were performed for each analysis.

### Analysis of gene expression using quantitative reverse transcription PCR

The leaves of bacterium-inoculated and un-inoculated tomato plants were sampled at 0/6 hours after a challenge by *S. litura* for analysis of the defense-related gene expression. Firstly, the leaves were ground to powder in liquid nitrogen, then total RNA was extracted using TRIzol reagent (TaKaRa, Japan). The first-strand cDNA was synthesized from 1000 ng of total RNA using GoScript™ Reverse Transcription Mix, Oligo (dT) according to the manufacturer’s instructions. The reverse transcriptional reaction condition was: 25 °C, 5 min; 42 °C, 60 min; 75 °C, 15 min; 4 °C, ∞. The quantitative reverse transcription PCR (RT-qPCR) experiments were performed to analyze the expression levels of genes encoding allene oxide cyclase (*AOC*)*,* allene oxide synthase (*AOS*)*,* lipoxygenase D (*LOXD*) and proteinase inhibitor (*PI-II*). The gene-specific primers used in the study were listed in the Table S2 [27]. The RT-qPCR reactions were carried out with 12.5 μL of the SYBR green master mix, 1 μL cDNA, 0.2 μL (10 μM) of each specific primer, and 11 μL RNase free water. RT-qPCR was performed in Step One Plus PCR instrument (Applied Biosystems). The thermal cycle reaction condition was: 95 °C,1 min; 95 °C,20 s; 58–60 °C,15 s;72 °C,30 s; 40 cycles; 82 °C,1 s. The housekeeping gene actin was used as an endogenous control in the RT-qPCR experiment.

### Influence of T6–4 isolate inoculation on tomato growth

Growth medium of around the roots of tomato plants was inoculated with the suspension of T6–4 isolate (OD600 ≈ 0.8). An equal volume of distilled water was added instead of the suspension of T6–4 isolate in the control. Each plant received 1000 μL bacterial suspension of T6–4 isolate or distilled water twice a week in the greenhouse condition. Each treatment had 20 plants. After 30 days the growth traits including shoot length, length of the longest leaf, shoot fresh weight and dry weight were measured to evaluate the effects of the bacterial inoculation on tomato growth.

### Data analysis

**Fig. 1:**
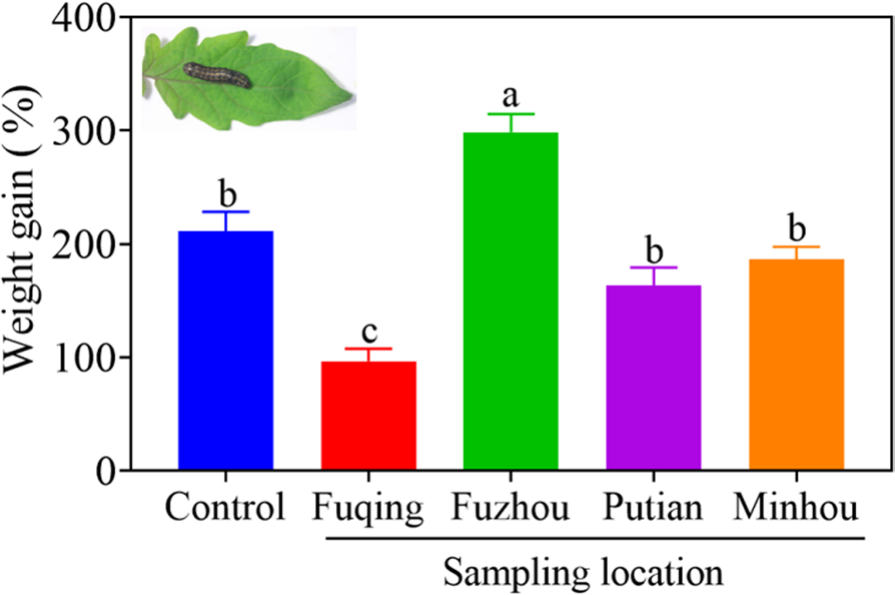
Weight gain of *S. litura* larvae fed on tomato leaves. Tomato plants were grown in sterilized soil inoculated with the microbes from soils collected from four locations in Fujian Province, China, and inoculated with *S. litura* larvae (see Materials and Methods). Control larvae are those fed on the plants without microbial inoculation. Values are mean ± SE (*n* = 30). The letters above the bars indicate the significant differences among treatments (one-way ANOVA and Tukey’s multiple range test, *P* < 0.05)

Graphpad Prism 8.0.2 software and Microsoft Excel 2013 were used to process and plot the data. SPSS 19 (SPSS, Chicago, IL, USA) software was used for statistical analysis [38]. The bioassays, physiological and biochemical experiments were performed by a completely randomized design. A one-way ANOVA and Tukey’s multiple range test (*P* < 0.05) were used to evaluate the significance of differences among different treatments (Fig. 1). The independent sample T-test was used to evaluate the significance of differences between the bacterium-inoculated group and the un-inoculated group. The normality (*P* > 0.05) of all data and homogeneity of variance (*P* > 0.05) were confirmed through Shapiro-Wilk test and Levene’s test, respectively using SPSS 19 software.

## Results

### Effect of bacteria in rhizosphere soils from different locations on tomato anti-herbivore resistance

The soil slurries obtained from Fuqing and Fuzhou showed obvious effects on tomato resistance against *S. litura*, while the others obtained from Putian and Minhou showed no significant effect (Fig. 1). Weight gain of *S. litura* larvae fed on the leaves of tomato plants inoculated with the bacteria in the rhizosphere from Fuqing location was significantly lower than those from the other three locations that were decreased by 114% relative to control. On the contrary, *S. litura* larvae fed on tomato plants inoculated with the bacteria in the rhizosphere from Fuzhou location showed an 88% increase compared with the control, i.e., without bacterial inoculation.

### Isolation and screening of rhizosphere bacteria that induces tomato anti-herbivore resistance

**Fig. 2:**
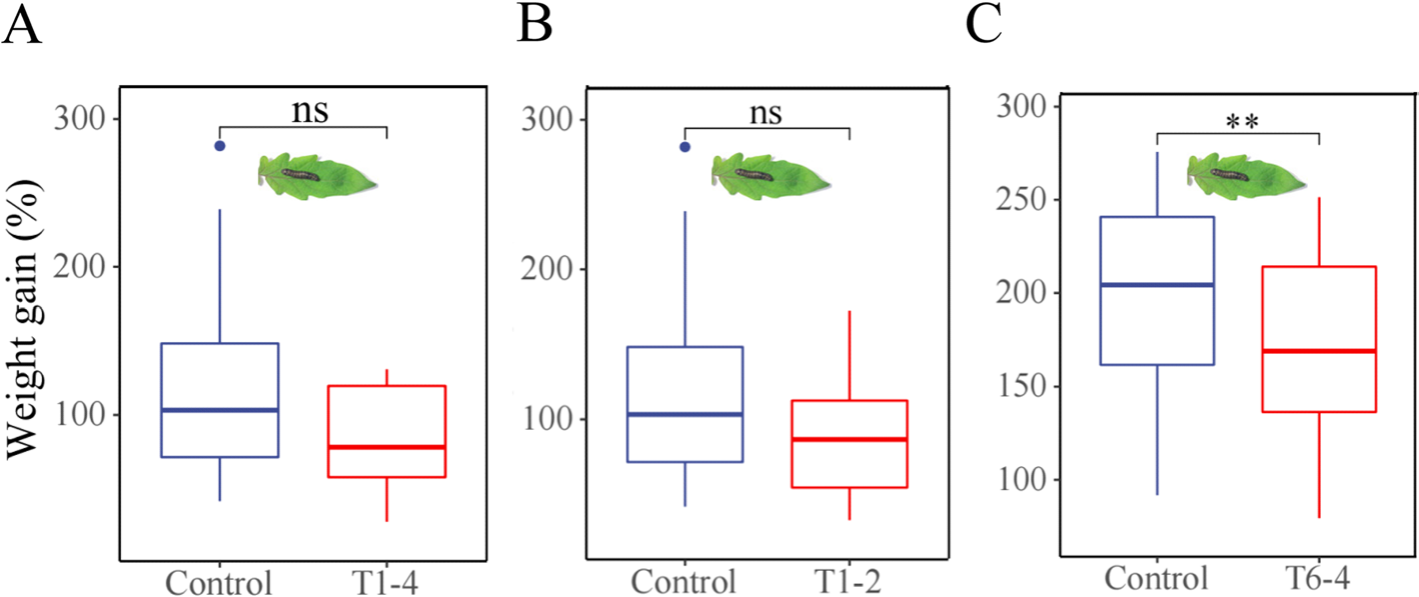
Weight gain of *S. litura* larvae fed on tomato plants grown in soil inoculated with rhizosphere bacteria isolates. Three isolates, T1–4 (**A**), T1–2 (**B**) and T6–4 (**C**), were isolated from soil collected from Fuqing in Fujian Province, China. Control larvae are those that were fed on the plants without microbial inoculation. Values are mean ± SE (*n* = 60). The asterisks indicate statistically significant differences according to the independent t-test between the bacteria treated group and the control-treated group (**P < 0.01). ns, not significant

Based on the results above, the soil of the rhizosphere from Fuqing was subjected to further screening and isolation of the bacteria that induced tomato anti-herbivore resistance. A total of 102 isolates were obtained from the rhizosphere of tomato plants. Among these isolates, three of them, designated as T1–4, T1–2 and T6–4, showed some effects on tomato resistance against *S. litura* (Fig. 2A, B and C). The effects of other isolates including individual isolates and mixture isolates used in the study on the weight gains of *S. litura* were shown in Table S3–1 and Table S3–2. Particularly, the weight gain of the larvae fed on the tomato plants inoculated with T6–4 isolate decreased by 27% (*p* = 0.0045) relative to control (Fig. 2C).

### Identification of the bacterial T6–4 isolate

**Fig. 3:**
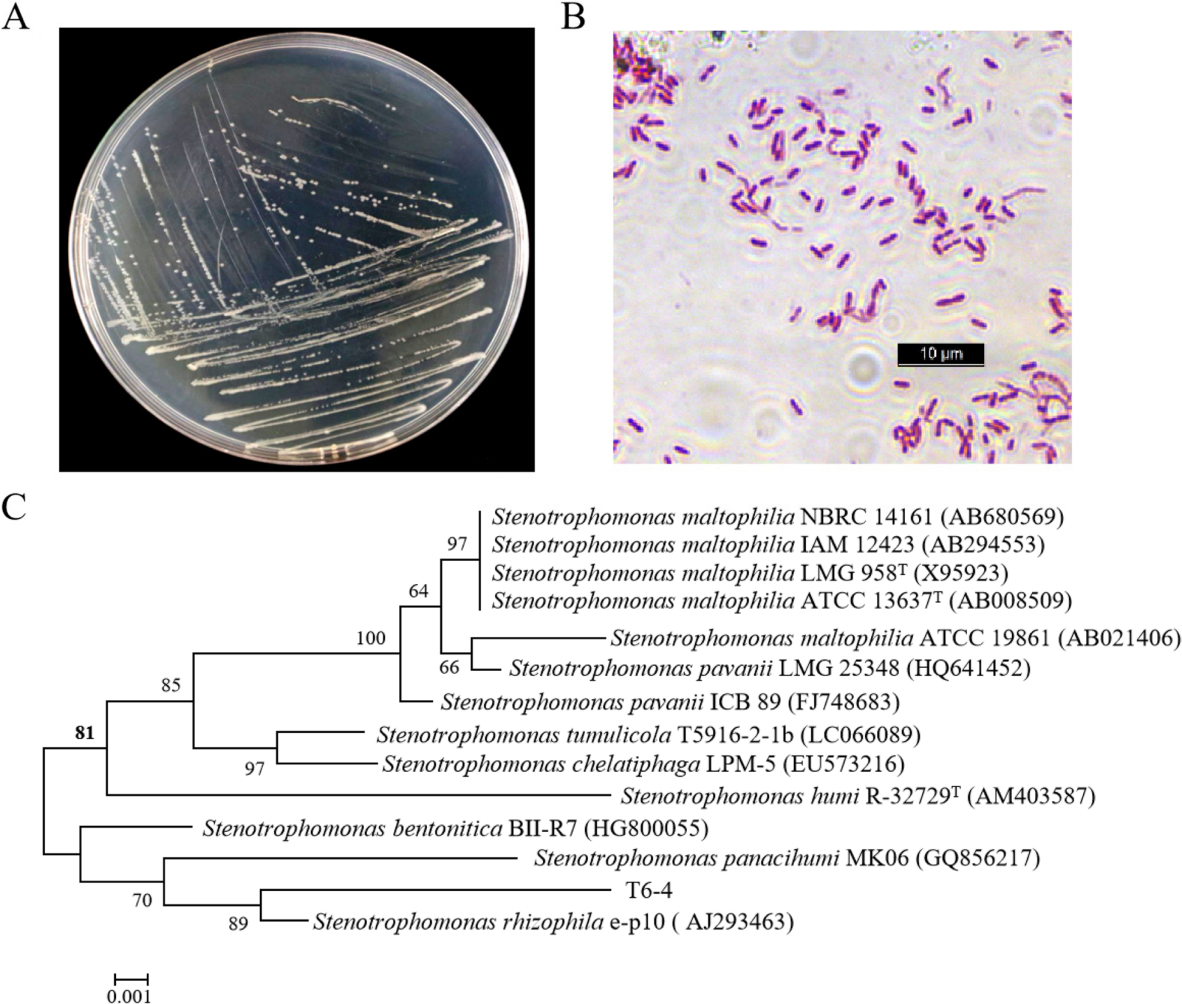
Identification of the T6–4 isolate. **A** Morphological characteristics of T6–4 isolate on TSA culture medium. **B** Cellular morphology of the isolate observed under the microscope. **C** Neighbor-joining phylogenetic tree of isolate T6–4 based on the 16S rRNA gene sequence. Bootstrap percentage values based on 1000 replications are listed at the branches and only values greater than 60% are shown at the nodes of the tree. Bar, 0.001 substitutions per nucleotide base

The morphological characteristics of the T6–4 isolate were determined. The isolate T6–4 cultured on the TSA medium plate at 28 °C for 24 h displayed the spherical, smooth, convex, and primrose yellow colony (Fig. 3A). The gram staining technique was then used to identify the isolate. The cellular morphology of the isolate was observed with a microscope. The shape of bacterial cells appears to be a straight or slightly curved rod with a size of 2.5 μm, suggesting that it belongs to a gram-negative bacterium (Fig. 3B). The 16S rRNA gene sequence containing 1456 bp was obtained and a neighbor-joining phylogenetic tree was constructed (Fig. 3C). The sequence analysis indicated that the isolate T6–4 was closely related to *Stenotrophomonas rhizophila* with 99.37% sequence similarity. The isolate was defined as *Stenotrophomonas* sp. T6–4 (Fig. 3C).

### Induction of protease inhibitor (PI) and defense-related enzyme activity by T6–4 isolate

**Fig. 4:**
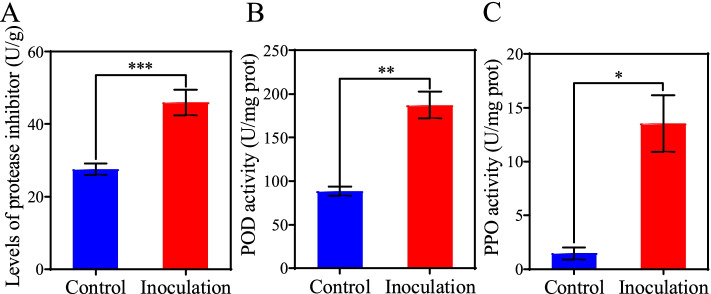
Enzyme activities in tomato levels. The activities of protease inhibitor (PI, A), peroxidase (POD, **B**) and polyphenol oxidase (PPO, **C**) were determined using the leaves from tomato plants grown in sterilized soil inoculated with *Stenotrophomonas* sp. T6–4 and 24 h after *S. litura* larvae inoculation. Values are means ± SE from three replicates. The asterisk indicates statistically significant differences according to independent t-test between the bacterium-treated group and control-treated group (*P < 0.05,**P < 0.01,***P < 0.001)

Protease inhibitors (PIs) play a vital role in tomato defense against insect pests [34]. Inoculation with *S. rhizophila* T6–4 isolate led to a 66.9% increase in PI activity in tomato leaves (Fig. 4 A). Similarly, the activities of the defense-related enzymes POD and PPO in the leaves of tomato plants inoculated with T6–4 isolate were significantly increased by 53 and 80%, respectively, as compared to the un-inoculated control (Fig. 4B & C).

### Induction of defense-related genes by T6–4 isolate

**Fig. 5:**
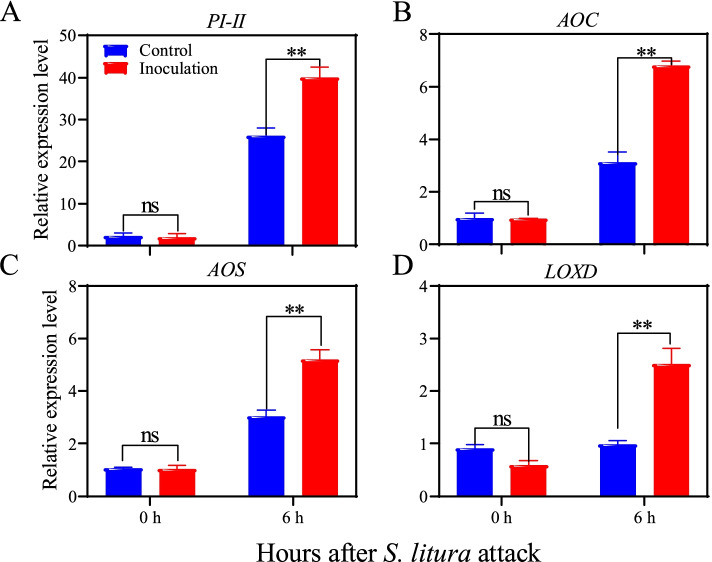
RT-qPCR analysis of defense-related genes in tomato plants. Total RNA was isolated from the leaves of tomato plants grown in the soil inoculated with T6–4 isolate and *S. litura* larvae. Four genes were selected and subjected RT-qPCR using gene-specific primers. These genes included (**A**) proteinase inhibitor (*PI-II*), (**B**) allene oxide cyclase (*AOC*), (**C**) allene oxide synthase (*AOS*), and (**D**) lipoxygenase D (*LOXD*). Values are means ± SE from three replicates. The asterisks indicate statistically significant differences according to the independent t-test between the bacterium-inoculated group and the control group (**P < 0.01). ns, no significant difference

To investigate the impact of T6–4 isolate on the gene expression in tomato plants, four defense-related genes, i.e., allene oxide cyclase (*AOC*), allene oxide synthase (*AOS*), lipoxygenase D (*LOXD*) and proteinase inhibitor (*PI-II*) were selected and analyzed by RT-qPCR. As shown in Fig. 5, inoculation with T6–4 isolate in tomato rhizosphere induced the expression of all four defense-related genes 6 h after insect inoculation. The relative expression level of *PI-II* in the leaves of tomato plants inoculated with T6–4 isolate was 1.4 fold higher than control (Fig. 5A), while the jasmonate biosynthesis genes, *AOC, AOS* and *LOXD*, in tomato leaves were 2.2-, 1.7- and 2.7-fold, respectively (Fig. 5B, C, D).

### Impact of the T6–4 isolate on tomato growth

**Fig. 6:**
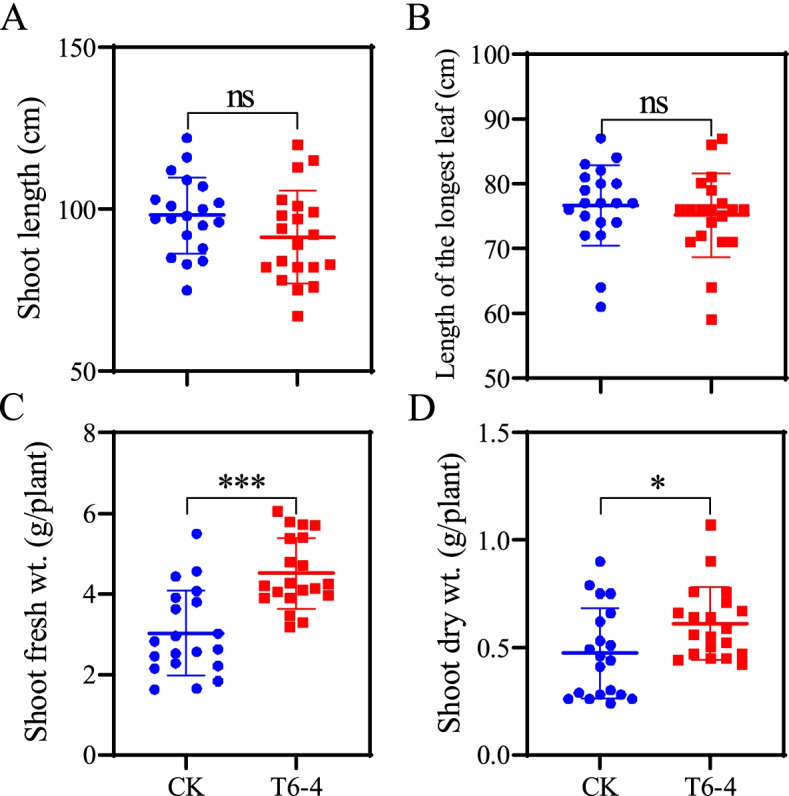
Effects of inoculation with T6–4 isolate on the tomato growth. Shoot length (**A**), length of the longest leaf (**B**), shoot fresh weight (**C**) and dry weight (**D**) were measured 30 days after bacterial inoculation. Control plants received an equal volume of distilled water. Values are means ± SE (*n* = 20). The asterisks indicate statistically significant differences according to independent t-test between the bacterium-inoculated group and control group (*P < 0.05, **P < 0.01, ***P < 0.001). ns, not significant

As compared to the control, inoculation with T6–4 isolate did not significantly affect the lengths of the shoot and the longest leaf (Fig. 6A & B). However, the bacterial inoculation increased shoot fresh weight and dry weight by 33 and 23%, respectively (Fig. 6C & D).

## Discussion

The enormous diversity of rhizosphere microbes, also referred to as the second genome of the plant, plays an important role in plant resistance against insect herbivores and microbial pathogens [9, 31, 39, 40]. Plant health is highly dependent on its rhizosphere microbes [41, 42]. Recent evidence indicates that plants are capable of recruiting certain beneficial rhizosphere microbes to suppress pathogens in the rhizosphere [40, 43–45]. This study shows that the tomato rhizosphere harbors beneficial bacteria that induce plant anti-herbivore defense. Soil slurry from tomato rhizosphere collected from Fuqing enhanced tomato resistance against chewing caterpillar *S. litura* (Fig. 1). From the soil of the tomato rhizosphere, we isolated the bacterial isolate T6–4 that enhanced tomato anti-herbivore resistance (Fig. 2). Based on morphology and 16S rDNA sequence, the isolate T6–4 was identified as *Stenotrophomonas* sp. T6–4 (Fig. 3)*.*

Bacteria of the genus *Stenotrophomonas* have gained increasing interest due to their ubiquitous occurrence, suppression of disease and promotion of growth [46, 47]. *S. maltophilia* in the rhizosphere of oilseed rape shows in vitro and greenhouse inhibitory effects on the growth of *Rhizoctonia solani* and *Verticillium dahliae* var. longisporum, two common soil-borne pathogens of rape (*Brassica napus* L.) [48]*. S. rhizophila* is an active participant in the rhizosphere and endosphere, and it has the potential for use to promote plant growth and control plant diseases [49]. *S. rhizophila* is also considered as a salt-tolerant beneficial microorganism due to its production of glucosylglycerol (GG) and trehalose, which are compatible solutes that allow plants to acclimate to enhance salinities, desiccation, and cold stress [50]. In the highly salinated soils in Uzbekistan, the promotion of plant growth by *S. rhizophila* DSM14405^T^ is up to 180% [51]. Such plant growth promotion effect is particularly apparent in tomato plants [47]. *S. rhizophila* DSM14405^T^ stimulates plant growth by eliminating deleterious microorganisms in the soil [47, 52].

However, little is known about the role of the bacterial genus in plant anti-herbivore resistance. Our study showed that *S. litura* feeding on the leaves of tomato was affected by root colonization by *S. rhizophila* T6–4 isolate obtained from rhizosphere soil of tomato. Several recent studies have shown that inoculation with rhizosphere bacteria enhances plant defense against insect herbivores [3, 12, 53]. The bacteria were inoculated in the roots and their colonization enhanced anti-herbivore defense in the leaves, suggesting that systemic defense responses had been induced by those rhizosphere bacteria. A similar finding by Pangesti et al. showed that inoculation of *Arabidopsis* roots with rhizobacteria enhanced plant resistance against chewing insects though increased expression of JA-dependent gene *LOX2* [19]. Likewise, Zebelo et al. reported that rhizosphere bacteria enhanced cotton resistance against the leaf-chewing insect *S. exigua* by induction of JA-related genes and (+)-δ-cadinene synthase genes involved in the biosynthesis of gossypol [12]. Here we also found that upon *S. litura* infestation the expression levels of JA-related genes including *AOC, AOS, LOXD* and *PI-II* were induced by bacterial inoculation with T6–4 isolate (Fig. 5). The activities of defense-related enzymes and protease inhibitors were also enhanced by bacterial inoculation (Fig. 4). Similarly, Bano & Muqarab revealed that rhizobacteria-inoculated plants showed enhanced activities of antioxidant enzymes POD and SOD, polyphenol oxidases (PPO) and proteinase inhibitors, which contributed to increased protection of tomato plants against *S. litura* [3]. Accumulation of PIs has been considered as a plant defensive response to insect herbivores [54, 55]. Plant PIs can bind to the digestive enzymes in insect guts and inhibit their activity, thereby reduce protein digestion, resulting in slow development and/or starvation [16]. Inoculation with a combination of *Pseudomonad* isolates Pf1, TDK1 and PY15 induced accumulation of proteinase inhibitors that contributed to enhanced rice defense against leaffolder, *Cnaphalocrocis medinalis* Guen [56]. This study also showed that *Stenotrophomonas* sp. T6–4 improved tomato growth (Fig. 6C & D) as well as enhanced plant resistance against insect herbivore *S. litura*.

In conclusion, our findings demonstrate that tomato rhizosphere harbors some beneficial bacteria that can systemically induce JA-dependent defense responses, leading to enhanced plant resistance to chewing insect herbivore *S. litura*. This study reveals a novel approach of screening beneficial bacteria to induce plant anti-herbivore resistance. Our findings suggest that the use of soil beneficial microorganisms has great potential to control insect pests in agriculture.

## Supplementary Information

**Additional file 1.**

## Data Availability

The datasets generated and/or analyzed during the current study are available in the National Center for Biotechnology Information (NCBI) repository, accession No: OM536158. All data of this study are included in the published article. Correspondence and requests for materials should be addressed to Yuanyuan Song (yyuansong@fafu.edu.cn).

## Abbreviations

POD: Peroxidase
PPO: Polyphenol oxidase
PI: Protease inhibitors
*AOC*: Allene oxide cyclase gene
*AOS*: Allene oxide synthase gene
*LOXD*: Lipoxygenase gene
*PI-II*: Proteinase inhibitor gene

## References

[1] Giuntini D, Graziani G, Lercari B, Fogliano V, Soldatini GF, Ranieri A (2005). Changes in carotenoid and ascorbic acid contents in fruits of different tomato genotypes related to the depletion of UV-B radiation. J Agric Food Chem.

[2] Saito T, Takagi M, Tezuka T, Ogawara T, Wari D (2021). Augmenting *Nesidiocoris tenuis* (Nesidiocoris) with a factitious diet of *Artemia* cysts to control *Bemisia tabaci* (Gennadius) on tomato plants under greenhouse conditions. Insects.

[3] Bano A, Muqarab R (2017). Plant defence induced by PGPR against *Spodoptera litura* in tomato (*Solanum lycopersicum* L.). Plant Biol.

[4] Tong H, Su Q, Zhou X, Bai L (2013). Field resistance of Spodoptera litura (Lepidoptera: Noctuidae) to organophosphates, pyrethroids, carbamates and four newer chemistry insecticides in Hunan, China. J Pest Sci.

[5] Shad SA, Sayyed AH, Fazal S, Saleem MA, Zaka SM, Ali M (2012). Field evolved resistance to carbamates, organophosphates, pyrethroids, and new chemistry insecticides in *Spodoptera litura* fab. (Lepidoptera: Noctuidae) J Pest Sci.

[6] Dang K, Doggett SL, Veera Singham G, Lee CY (2017). Insecticide resistance and resistance mechanisms in bed bugs, *Cimex* spp. (Hemiptera: Cimicidae). Parasit Vectors.

[7] Heckel DG (2012). Insecticide resistance after silent spring. Science.

[8] Gould F, Brown ZS, Kuzma J (2018). Wicked evolution: can we address the sociobiological dilemma of pesticide resistance?. Science.

[9] Larousse M, Rancurel C, Syska C, Palero F, Etienne C, Industri B (2017). Tomato root microbiota and *Phytophthora parasitica*-associated disease. Microbiome.

[10] Yang JW, Yi HS, Kim H, Lee B, Lee S, Ghim SY (2011). Whitefly infestation of pepper plants elicits defence responses against bacterial pathogens in leaves and roots and changes the below ground micro-flora. J Ecol.

[11] Zahid M, Abbasi MK, Hameed S, Rahim N (2015). Isolation and identification of indigenous plant growth promoting rhizobacteria from Himalayan region of Kashmir and their effect on improving growth and nutrient contents of maize (*Zea mays* L.). Front Microbiol.

[12] Zebelo S, Song Y, Kloepper JW, Fadamiro H (2016). Rhizobacteria activates (+)-δ-cadinene synthase genes and induces systemic resistance in cotton against beet armyworm (*Spodoptera exigua*). Plant Cell Environ.

[13] Spaink H (2000). Root nodulation and infection factors produced by rhizobial bacteria. Annu Rev Microbiol.

[14] Dobbelaere S, Vanderleyden J, Okon Y (2010). Plant growth promoting effects of diazotrophsin the rhizosphere. Crit Rev Plant Sci.

[15] Frost CJ, Mescher MC, Carlson JE, De Moraes CM (2008). Plant defense priming against herbivores: getting ready for a different battle. Plant Physiol.

[16] War AR, Paulraj MG, Ahmad T, Buhroo AA, Hussain B, Ignacimuthu S (2012). Mechanisms of plant defense against insect herbivores. Plant Signal Behav.

[17] Maffei ME, Mithöfer A, Boland W (2007). Insects feeding on plants: rapid signals and responses preceding the induction of phytochemical release. Phytochemistry.

[18] Kessler A, Halitschke R, Baldwin IT (2004). Silencing the Jasmonate Cascade: induced plant defenses and insect populations. Science.

[19] Pangesti N, Pineda A, Dicke M, Van Loon JJA (2015). Variation in plant-mediated interactions between rhizobacteria and caterpillars: potential role of soil composition. Plant Biol.

[20] Ahn IP, Lee SW, Suh SC (2007). Rhizobacteria-induced priming in Arabidopsis is dependent on ethylene, jasmonic acid, and NPR1. Mol Plant-Microbe Interact.

[21] Valenzuela-Soto JH, Estrada-Hernández MG, Ibarra-Laclette E, DélanoFrier JP (2010). Inoculation of tomato plants (*Solanum lycopersicum*) with growth-promoting *Bacillus subtilis* retards whitefly *Bemisia tabaci* development. Planta.

[22] Rashid MH, Chung YR (2017). Induction of systemic resistance against insect herbivores in plants by beneficial soil microbes. Front Plant Sci.

[23] Dunse KM, Stevens JA, Lay FT, Gaspar YM, Heath RL, Anderson MA (2010). Coexpression of potato type I and II proteinase inhibitors gives cotton plants protection against insect damage in the field. Proc Natl Acad Sci U S A.

[24] Azzouz H, Cherqui A, Campan EDM, Rahbé Y, Duport G, Jouanin L (2005). Effects of plant protease inhibitors, oryzacystatin I and soybean BowmanBirk inhibitor, on the aphid *Macrosiphum euphorbiae* (Homoptera, Aphididae) and its parasitoid *Aphelinus abdominalis* (Hymenoptera, Aphelinidae). J Insect Physiol.

[25] Qiao JQ, Yu X, Liang XJ, Liu YF, Borriss R, Liu YZ (2017). Addition of plant-growth-promoting *Bacillus subtilis* PTS-394 on tomato rhizosphere has no durable impact on composition of root microbiome. BMC Microbiol.

[26] Hanafi A, Traoré M, Schnitzler WH, Woitke M (2007). Induced resistance of tomato to whiteflies and *Phytium* with the PGPR *Bacillus subtilis* in a soilless crop grown under greenhouse conditions. Acta Horticul.

[27] Song YY, Ye M, Li CY, He X, Zhu-Salzman K, Wang RL (2014). Hijacking common mycorrhizal networks for herbivore-induced defence signal transfer between tomato plants. Sci Rep.

[28] Gupta GP, Rani S, Birah A, Raghuraman M (2005). Improved artificial diet for mass rearing of the tobacco caterpillar, *Spodoptera litura* (Lepidoptera: Noctuidae). Inter J Trop Insect Sci.

[29] Smalla K, Wieland G, Buchner A, Zock A, Parzy J, Kaiser S (2001). Bulk and rhizosphere soil bacterial communities studied by denaturing gradient gel electrophoresis: plant-dependent enrichment and seasonal shifts revealed. Appl Environ Microbiol.

[30] Kostenko O, Van de Voorde TFJ, Mulder PPJ, Van der Putten WH, Bezemer TM (2012). Legacy effects of aboveground-belowground interactions. Ecol Lett.

[31] Yuan J, Zhao J, Wen T, Zhao ML, Li R, Goossens P (2018). Root exudates drive the soil-borne legacy of aboveground pathogen infection. Microbiome.

[32] Gottel N, Castro HF, Kerley M, Yang Z, Pelletier DA, Podar M (2011). Distinct microbial communities within the endosphere and rhizosphere of *Populus deltoides* roots across contrasting soil types. Appl Environ Microbiol.

[33] Zhang J, Liu JY, Meng LY, Ma ZY, Tang XY, Cao YY (2012). Isolation and characterization of plant growth-promoting rhizobacteria from wheat roots by wheat germ agglutinin labeled with fluorescein isothiocyanate. J Microbiol.

[34] Park M, Kim C, Yang J, Lee H, Shin W, Kim S (2005). Isolation and characterization of diazotrophic growth promoting bacteria from rhizosphere of agricultural crops of Korea. Microbiol Res.

[35] Long HH, Sonntag DG, Schmidt DD, Baldwin IT (2010). The structure of the culturable root bacterial endophyte community of *Nicotiana attenuata* is organized by soil composition and host plant ethylene production and perception. New Phytol.

[36] Han Y, Li P, Gong S, Yang L, Wen L, Hou M (2016). Defense responses in rice induced by silicon amendment against infestation by the leaf folder *Cnaphalocrocis medinalis*. Plos One.

[37] Neog M, Saikia L (2010). Control of post-harvest pericarp browning of litchi (*litchi chinensis Sonn*). J Food Sci Technol.

[38] Zheng YQ, Zhang XY, Liu X, Qin NN, Xu KF, Zeng RS (2021). Nitrogen supply alters rice defense against the striped stem borer *Chilo suppressalis*. Front Plant Sci.

[39] Lareen A, Burton F, Schäfer P (2016). Plant root-microbe communication in shaping root microbiomes. Plant Mol Biol.

[40] Hu L, Robert CAM, Cadot S, Zhang X, Ye M, Li B (2018). Root exudate metabolites drive plant-soil feedbacks on growth and defense by shaping the rhizosphere microbiota. Nat Commun.

[41] Berendsen RL, Pieterse CM, Bakker PA (2012). The rhizosphere microbiome and plant health. Trends in Plant Sci.

[42] Trivedi P, Leach JE, Tringe SG, Sa T, Singh BK (2020). Plant-microbiome interactions: from community assembly to plant health. Nat Rev Microbiol.

[43] Kwak MJ, Kong HG, Choi K, Kwon SK, Song JY, Lee J (2018). Rhizosphere microbiome structure alters to enable wilt resistance in tomato. Nat Biotechnol.

[44] Carrión VJ, Perez-Jaramillo J, Cordovez V, Tracanna V, De Hollander M, Ruiz-Buck D (2019). Pathogen-induced activation of disease-suppressive functions in the endophytic root microbiome. Science.

[45] Shi Y, Pan Y, Xiang L, Zhu Z, Fu W, Hao G (2022). Assembly of rhizosphere microbial communities in *Artemisia annua*: recruitment of plant growth-promoting microorganisms and inter-kingdom interactions between bacteria and fungi. Plant Soil.

[46] Messiha NAS, Van Diepeningen AD, Farag NS, Abdallah SA, Janse JD, Van Bruggen AHC (2007). *Stenotrophomonas maltophilia*: a new potential biocontrol agent of *Ralstonia solanacearum*, causal agent of potato brown rot. Eur J Plant Pathol.

[47] Schmidt CS, Alavi M, Cardinale M, Müller H, Berg G (2012). *Stenotrophomonas rhizophila* DSM14405^T^ promotes plant growth probably by altering fungal communities in the rhizosphere. Biol Fertil Soils.

[48] Berg G, Marten P, Ballin G (1996). *Stenotrophomonas maltophilia* in the rhizosphere of oilseed rape - occurrence, characterization and interaction with phytopathogenic fungi. Microbiol Res.

[49] Ryan R, Monchy S, Cardinale M, Taghavi S, Crossman L, Avison M (2009). The versatility and adaptation of bacteria from the genus *Stenotrophomonas*. Nat. Rev. Microbiol.

[50] Hagemann M, Ribbeck-Busch K, Klähn S, Hasse D, Steinbruch R, Berg G (2008). The plant-associated bacterium *Stenotrophomonas rhizophila* expresses a new enzyme for the synthesis of the compatible solute glucosylglycerol. J Bacteriol.

[51] Egamberdieva D, Kucharova Z, Davranov K, Berg G, Makarova N, Azarova T (2011). Bacteria able to control foot and root rot and to promote growth of cucumber in salinated soils. Biol Fertil Soils.

[52] Alavi P, Starcher M, Zachow C, Müller H, Berg G (2013). Root-microbe systems: the effect and mode of interaction of stress protecting agent (SPA) *Stenotrophomonas rhizophila* DSM14405^T^. Front Plant Sci.

[53] Pangesti N, Reichelt M, van de Mortel JE, Kapsomenou E, Gershenzon J, van Loon JJ (2016). Jasmonic acid and ethylene signaling pathways regulate glucosinolate levels in plants during rhizobacteria-induced systemic resistance against a leaf-chewing herbivore. J Chem Ecol.

[54] Fan Y, Yang W, Yan Q, Chen C, Li J (2020). Genome-wide identification and expression analysis of the protease inhibitor gene families in tomato. Genes.

[55] Casaretto JA, Corcuera LJ (1998). Proteinase inhibitor accumulation in aphid-infested barley leaves. Phytochemistry.

[56] Saravanakumar D, Muthumeena K, Lavanya N, Suresh S, Rajendran L, Raguchander T (2007). *Pseudomonas*-induced defence molecules in rice plants against leaffolder (*Cnaphalocrocis medinalis*) pest. Pest Manag Sci.

