# Supplementary material for: Enhanced anti-herbivore defense of tomato plants against Spodoptera litura by their rhizosphere bacteria
Source: BMC Plant Biol. 2022 May 24;22:254. doi: 10.1186/s12870-022-03644-3 (PMC9128215; doi:10.1186/s12870-022-03644-3)
Supplement: Supplementary file 1 — Additional file 1. [file 12870_2022_3644_MOESM1_ESM.doc]

| Culture media | Nutritional formula |
| --- | --- |
| Tryptic soy agar (TSA) | 15 g Tryptone, 5 g soybean peptone, 5 g NaCl, and 15 g agar |
| Lysogeny broth (LB) | 10 g tryptone, 5 g yeast powder, 5 g NaCl, and 15 g agar |
| Nutrient agar (NA) | 10 g tryptone, 3 g beef extract powder, 5 g NaCl, and 15 g agar |

### **Supporting material**

**Tab. S1** Nutritional formula of different bacterium media

**Tab. S2** Primers for RT-qPCR

| gene | Sequence of Primer |
| --- | --- |
| *AOC* | F: 5’-CTCGGAGATCTTGTCCCCTTT-3’  R: 5’-CTCCTTTCTTCTCTTCTTCGTGCT-3’ |
| *AOS* | F: 5’-CGATTACCTCCGATTCTGGT-3’  R: 5’-AAATCTTCATCCCACCGAAG-3’ |
| *LOXD* | F: 5’-CCGTGGTTGACACATTATCG-3’  R: 5’-ACAGCAGTCCGCCCTATTTA-3’ |
| *PI-Ⅱ* | F: 5’-AATTATCCATCATGGCTGTTCAC-3’  R: 5’-CCTTTTTGGATCAGATTCTCCTT-3’ |
| *Actin* | F: 5’-TGGTCGGAATGGGACAGAAG-3’  R: 5’-CTCAGTCAGGAGAACAGGGT-3’ |

**Tab. S3-1** Effects of individual isolate on the weight gains of *S. litura*

| Treatments | Weight gains of *S. litura* (mg) |
| --- | --- |
| Control | 19.83±1.39^a^ |
| NA | 20.45±1.09^a^ |
| T7-4 | 21.82±1.74^a^ |
| T8-3 | 17.57±1.16^a^ |

**Tab. S3-2** Effects of mixture isolates on the weight gains of *S. litura*

| Treatments | Weight gains of *S. litura* (mg) |
| --- | --- |
| Control | 42.85±2.50^a^ |
| F3-3-1/F1-1 | 41.86±3.22^a^ |
| F7-2/F2-3 | 38.87±2.61^a^ |
| T1-2/T2-4 | 41.20±2.64^a^ |

“a” means no significant differences among treatments (one-way ANOVA and Tukey’s multiple range test, *P* < 0.05).
